# Enhanced extraction of bioactive compounds from propolis (*Apis mellifera* L.) using subcritical water

**DOI:** 10.1038/s41598-023-42418-1

**Published:** 2023-09-12

**Authors:** Su-Bin Shin, Jin-Kyoung Lee, Min-Jung Ko

**Affiliations:** 1 Department of Food Science and Biotechnology, Global K-Food Research Center, Hankyong National University, Anseong-si, 17579 South Korea; 2 CPR S&T., Ltd., Gunpo-si, 15880 South Korea

**Keywords:** Engineering, Environmental biotechnology

## Abstract

The bioactive compounds and antioxidant activities of propolis extracts were investigated using subcritical water extraction (SWE). SWE was performed by varying temperature (110–200 °C) and time (10–30 min). SWE using only water as solvent successfully to extracted bioactive compounds from propolis using high-purity glass thimbles. The concentrations of galangin (16.37 ± 0.61 mg/g), and chrysin (7.66 ± 0.64 mg/g) were maximal at 200 °C for 20 min, and 170 °C for 20 min, respectively. The antioxidative properties from propolis increased with the increasing extraction temperature and extraction time on SWE. The maximum yields of the total phenolics (226.37 ± 4.37 mg/g), flavonoids (70.28 ± 1.33 mg/g), and antioxidant activities (88.73 ± 0.58%, 98.86 ± 0.69%, and 858.89 ± 11.48 mg/g) were obtained at 200 °C for 20 min. Compared with using ethanol extraction (at 25 °C for 24 h, total phenolics = 176.28 ± 0.35, flavonoids = 56.41 ± 0.65, antioxidant activities = 72.74 ± 0.41%, 95.18 ± 0.11%, 619.51 ± 8.17 mg/g), all yields of SWE extracts obtained at 200 °C for 20 min were higher. SWE is suitable for a much faster and more efficient method extracting bioactive compounds from propolis compared to traditional extraction method.

## Introduction

Natural compounds, such as propolis (bee glue), have been increasingly used as food ingredients to improve health and prevent diseases as health functional foods^1^. Propolis *(Apis mellifera* L.) is a sticky and resinous, natural substance produced by honeybees from plants or flowers by mixing saliva with enzymes. It is generally composed of 50% resin, 30% wax, 10% essential oils, 5% pollen, and 5% other substances, including the derivatives of amino acids, phenolic compounds, and flavonoids^2^. Propolis has antioxidant, anti-inflammatory, antifungal, antibacterial, antiviral, antiparasitic, anticancer, and antitumor properties^3–6^.

Flavonoids are a type of phenolic compounds that have a C6–C3–C6 carbon skeleton structure formed by two benzene rings linked with each other through a central three-carbon chain. Flavonoids are high-level natural antioxidants because of their ability to reduce free radical formation and scavenge free radicals^7^. Flavonoids have many biological activities, including antioxidant, antiallergic, anti-inflammatory, anticarcinogenic, and antiproliferative effects^8^. Studies have shown that propolis contains high levels of bioflavonoids, such as galangin, chrysin, pinocembrin, pinobanksin, kaempferol, apigenin, quercetin, and other phenolics^9^. Flavonoids such as galangin, and chrysin are present at high concentrations in propolis. Galangin has biological activities, such as antioxidant, antibacterial, anticancer, antimutagenic, anticlastogenic, and metabolic enzyme-modulating activities, and can suppress the genotoxicity of chemicals^10^. Chrysin has biological activities, such as antioxidant, anti-inflammatory activities, and can treatment various degenerative disorders^11^. The quantity of flavonoids in food is used as a criterion to evaluate the quality of propolis, a highly antioxidant food^12^.

The propolis structure consists of more than 50% wax components tightly bound to more than tens of carbons. Since flavonoids are less-polar compounds in which they are tightly bound to dozens or more tightly bound carbon connective tissues, extraction is not easy if the temperature and solvent conditions are unsuitable. Propolis has low solubility in water because it is made up of a mixture of bioactive components, such as highly viscous resins and waxes. Therefore, in general, the use of ethanol is the traditional and most common method for extracting active compounds from propolis^13^. However, ethanol is toxic to the human body, non-environmentally friendly, and requires a long extraction time^14^. In addition, fat-soluble components, such as inactive resin and beeswax, that have low solubility in water, are extracted together, which is problematic for use in food. Microwave, ultrasonic, solid phase, and supercritical fluid extraction are also used to extract propolis; however, to our knowledge, the subcritical water extraction (SWE) of propolis has not been studied. Sun et al.^15^ reported subcritical propane extraction combined with ultrasound assisted ethanol extraction for extracting flavonoids and terpene. Ghavidel et al.^16^ approached in process intensification based on subcritical water. Subcritical water was used to provide propolis oil in water nanoemulsions. They focused on using the organic solvent as a solvent or subcritical water as nanoemulsions.

SWE is an alternative technique that effectively extracts less polar flavonoid compounds using only water for a short extraction time of < 30 min. Subcritical water as a solvent is maintained in a liquid state at a set temperature (100–374 °C) and high pressure (> 10 MPa) by a varying temperature-dependent dielectric constant (ε)^17^. With an increase in the temperature and pressure of water, subcritical water has a lower dielectric constant similar to less-polar organic solvents, such as methanol and ethanol, which weakens the hydrogen bonds for easy extraction^18^. SWE is environmentally friendly, uses only water instead of organic solvents, and is an effective technology for extracting phenolics and flavonoids quickly.

Therefore, the aim of this study was to extract bioactive compounds (phenolics, flavonoids) from propolis using SWE and determine the optimum extraction temperature (110–200 °C) and time (10–30 min). The effects of the SWE conditions on the antioxidant activities (DPPH (2,2-diphenyl-1-picrylhydrazyl)-radical-scavenging activity, ABTS (2,2′-azino-bis-3-ethylbenzthiazoline-6-sulphonic acid)-radical-scavenging activity and FRAP (ferry reducing antioxidant power) assay) of the extracts were also assessed. The yields and antioxidant activities of the SWE extracts from propolis were compared to that of conventional solvent extraction using ethanol to confirm efficiency of SWE. SWE technique was effective in extracting phenolics and flavonoids with antioxidant activity selectively from propolis. Especially, SWE using only water as the solvent successfully to extracted bioactive compounds from propolis using high-purity glass thimbles.

## Materials and methods

### Sample preparation

Propolis was obtained from CPR S&T Co. in January 2022. Resin was collected from bees in sabana uruguayense. The sample concentrate constituted 80% pure propolis (dry matter), 16% premium potable alcohol, and 4% distilled water. The samples were stored in a shaded location below 10 °C until analysis.

### Chemicals and reagents

Standard chemicals of galangin, chrysin, gallic acid (3,4,5-trihydroxybenzoic acid), quercetin (2-(3,4-dihydroxyphenyl)-3,5,7-trihydroxy-4H-1-benzopyran-4-one), DPPH, ABTS, TPTZ (2,4,6-tripyridyl-s-triazine), and Trolox (( ±)-6-hydroxy-2,5,7,8-tetramethylchroman-2-carboxylic acid) were purchased from Sigma–Aldrich (St. Louis, MO, USA).

The organic solvents methanol and *n*-hexane were purchased from Deoksan Pure Chemicals (Ansan, Gyeonggi-do, South Korea). HPLC (high performance liquid chromatography)-grade solvents, including acetonitrile, water, and formic acid, were purchased from Avantor Baker (Phillipsburg, NJ, USA) and Samchun Pure Chemicals (Pyeongtaek, Gyeonggi-do, South Korea).

### SWE method

**Figure 1:**
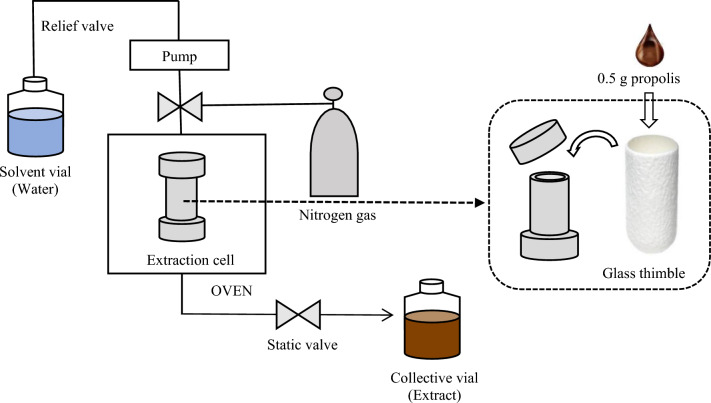
Schematic diagram of the SWE system using a glass thimble.

SWE was performed using an accelerated solvent extractor (ASE 350; Dionex, Sunnyvale, CA, USA) with purified water. In all experiments, propolis (0.5 g) was placed in a high-purity glass thimble filter (19 × 90 mm; Whatman, Maidstone, UK), and the thimble filter was placed in a stainless-steel extraction cell (22 mL, Dionex). Then, the extraction cell containing the thimbles with propolis was placed in a preheated oven, filled with 22 mL of purified water for approximately 30 s, and heated at a set temperature and time under high pressure (approximately 10 MPa). Extraction was performed at temperatures of 110 °C, 130 °C, 150 °C, 170 °C, 190 °C, and 200 °C for 10, 20, and 30 min in static mode without any outflow of solvent. The extract was purged with nitrogen gas for 1 min and collected in a vial. The extraction procedure is illustrated in Fig. 1. The collected extracts were frozen separately in a deep freezer and freeze-dried (Ilshin, Gyeonggi-do, South Korea) for 24 h. The dried sample was sealed and stored in a deep freezer at − 80 °C until analysis. All extractions were performed in triplicate under the same conditions.

### Conventional solvent extraction method

Conventional solvent extraction (CSE) was conducted to compare the SWE yields. Among the organic solvents, ethanol was used, and ethanol extraction is the most common extraction method used industrially to extract active compounds from resinous propolis^13^. 0.5 g of propolis was extracted using 22 mL of 50%, 70% and 100% ethanol at 25 °C for 24 h in the bath (C-WB, Changsin Scientific, Seoul, South Korea). After extraction, the ethanol solvent was removed using an evaporator (Rotavapor R-100; Buchi, Flawil, Switzerland) and the extract was solidified. The dried sample was sealed and stored in a deep freezer at − 80 °C until analysis. All experiments were repeated three times under the same conditions.

### HPLC analysis

The freeze-dried SWE extracts (0.05 g) were dissolved in 10 mL of methanol and then vortexed for 30 s by filtration through a 0.45 µm polyvinylidene fluoride filter (Whatman). Quantitative analysis of flavonoid compounds in the propolis extract was performed using a HPLC system (1200 series, Agilent Technologies, Santa Clara, CA, USA) with a Zorbax C18 column (4.6 × 250 mm, 5 μm pore size). The mobile phase consisted of solvent A for acetonitrile and solvent B for 0.1% formic acid in water. The gradient consisted of solvent A at the following concentrations: 40% for 0 min, 60% for 0–15 min, 60% for 15–30 min, and 40% for 30–40 min at a flow rate of 0.8 mL/min. The sample injection volume was 10 μL and the absorbance was monitored at 250 nm.

### Total phenolic contents analysis

The total phenolic contents (TPC) of SWE extracts were estimated using the method described by Hudz et al.^19^. 10 mg of freeze-dried extract was dissolved in 10 mL of 100% methanol and sonication was performed for 2 h. 0.1 mL of sample was mixed with 2 mL of 2% NaCl and vortexed for 3 min. 0.1 mL of 50% Folin–Ciocalteu reagent was added, and the mixture was left for 30 min. The absorbance of the TPC was measured at 700 nm using a UV–Vis spectrophotometer (Mega-U6000, Scinco, Seoul, South Korea), and the standard curve was constructed using the gallic acid standard. The TPC was expressed in milligrams of gallic acid equivalent per gram (mg GAE/g).

### Total flavonoid contents analysis

The total flavonoid contents (TFC) of the SWE extracts were estimated as described by Chang et al.^20^. 10 mg of freeze-dried extracts was dissolved in 4 mL of 100% methanol and sonication was performed for 2 h. Then, 0.5 mL of sample was mixed with 1.5 mL of 95% ethanol, and 0.1 mL of 10% aluminum chloride, 0.1 mL of 1 M potassium acetate, and 2.8 mL of distilled water were added, and the mixture was left for 30 min. A UV–Vis spectrophotometer (Scinco) was used at a wavelength of 415 nm to measure the absorption of TFC, and a standard curve was constructed using a quercetin standard. The TFC were expressed as milligrams of quercetin equivalents per gram (mg QE/g).

### DPPH free radical scavenging activity

DPPH radical scavenging activity of the SWE extracts was estimated using the method described by Maity et al.^21^. 10 mg of freeze-dried extracts was dissolved in 10 mL of 100% methanol, and sonication was performed for 2 h. 0.2 mL of sample was dissolved in 1 mL of 100 μM DPPH reagent and the mixture was left for 15 min. A UV–Vis spectrophotometer (Scinco) was used at a wavelength of 517 nm to measure the DPPH radical scavenging activity. The proportion of inhibition (%) was calculated using the following equation: (1 − A/B) × 100, where B is the absorbance of the blank and A is the absorbance of the samples.

### ABTS free radical scavenging activity

ABTS radical cation decolorization assay of the SWE extracts was estimated as described by Abbas et al.^22^. 10 mg of freeze-dried extract were dissolved in 10 mL of 100% methanol and sonication was performed for 2 h. Before the assay, 7 mM ABTS was added to 2.45 mM potassium persulfate to make ABTS reagent. The mixture was allowed to stand at room temperature in the dark for 16 h and then diluted with anhydrous ethanol to 0.7 at 734 nm. Then, 0.1 mL of the sample was mixed with 0.9 mL of ABTS reagents and allowed to stand for 6 min. A UV–Vis spectrophotometer (Scinco) was used at a wavelength of 735 nm to measure the ABTS free radical scavenging activity. The proportion of inhibition (%) was calculated using the same equation as that for the DPPH free radical scavenging activity.

### FRAP assay

FRAP assay of SWE extracts was performed using the method described by Hajri et al.^23^. The reagent was formed of 300 mM acetate buffer (3.1 g of C_2_H_3_NaO_2_·3H_2_O and 16 mL of C_2_H_4_O_2_), 10 mM trypyridyltriazine (TPTZ) solution (3.1233 g TPTZ in 40 mM HCl), and 20 mM FeCl_3_·6H_2_O solution. Then, the measurement sample was formed by mixing 0.5 g of sample with a 20 mL mixture of acetone and water at a ratio of 1:1, and the reagent was prepared by mixing 25 mL of 300 mM acetate buffer (pH 3.6), 2.5 mL of 10 mM TPTZ reagent, and 2.5 mL of 20 mM FeCl3·6H2O reagent, and preheated at 37 °C on the day of use. 10 μL of the sample was dissolved in 300 μL of the FRAP solution, left for 5 min in a dark room, and the absorption of the FRAP assay was measured at a wavelength of 593 nm using the UV–Vis spectrophotometer (Scinco). Absorption was calculated from a standard curve using the Trolox standard. FRAP was expressed as milligrams of Trolox equivalents per gram (mg TE/g).

### Data analysis

The extraction yield was calculated from the calibration curve of standard compounds. The optimum temperature and time for SWE of propolis were chosen based on the highest concentration or activity. All data are presented as the mean ± standard deviation of three measurements. The statistical analysis was performed a one-way analysis of variance with significant variation at *p* < 0.05 in Duncan’s test, and Pearson’s correlation analysis with significant variation at *p* < 0.01 using SPSS statistical software (version 21, IBM, Chicago, IL, USA).

## Results and discussion

### SWE technique for extracting bioactive compounds

The water solubility of propolis is low because of the resin material present, which accounts for the largest proportion of propolis, and is mostly composed of less polar phenolics and flavonoids. By increasing the temperature and pressure of water during SWE, subcritical water forms a lower dielectric constant (ε = 35 at 200 °C) like organic solvents, such as ethanol (ε = 25.3 at 20 °C)^16^. A low dielectric constant indicates that subcritical water weakens hydrogen bonds and less-polar compounds can be extracted easily. Using the SWE technique, the less-polar bioactive compounds such as phenolics and flavonoids in propolis could be extracted using only purified water.

Another factor in the success of propolis extraction with only pure water without organic solvent is the using high purity glass thimbles filter made of 100% borosilicate glass that has heat resistance up to 500 °C (Fig. 1). The high-purity glass thimble filter prevents impurities, such as resin and beeswax, from entering extracts, and entering the extract line of the machine. SWE was successful in extracting high concentration bioactive compounds without impurities from propolis with only purified water.

### Optimal SWE conditions of galangin and chrysin

**Figure 2:**
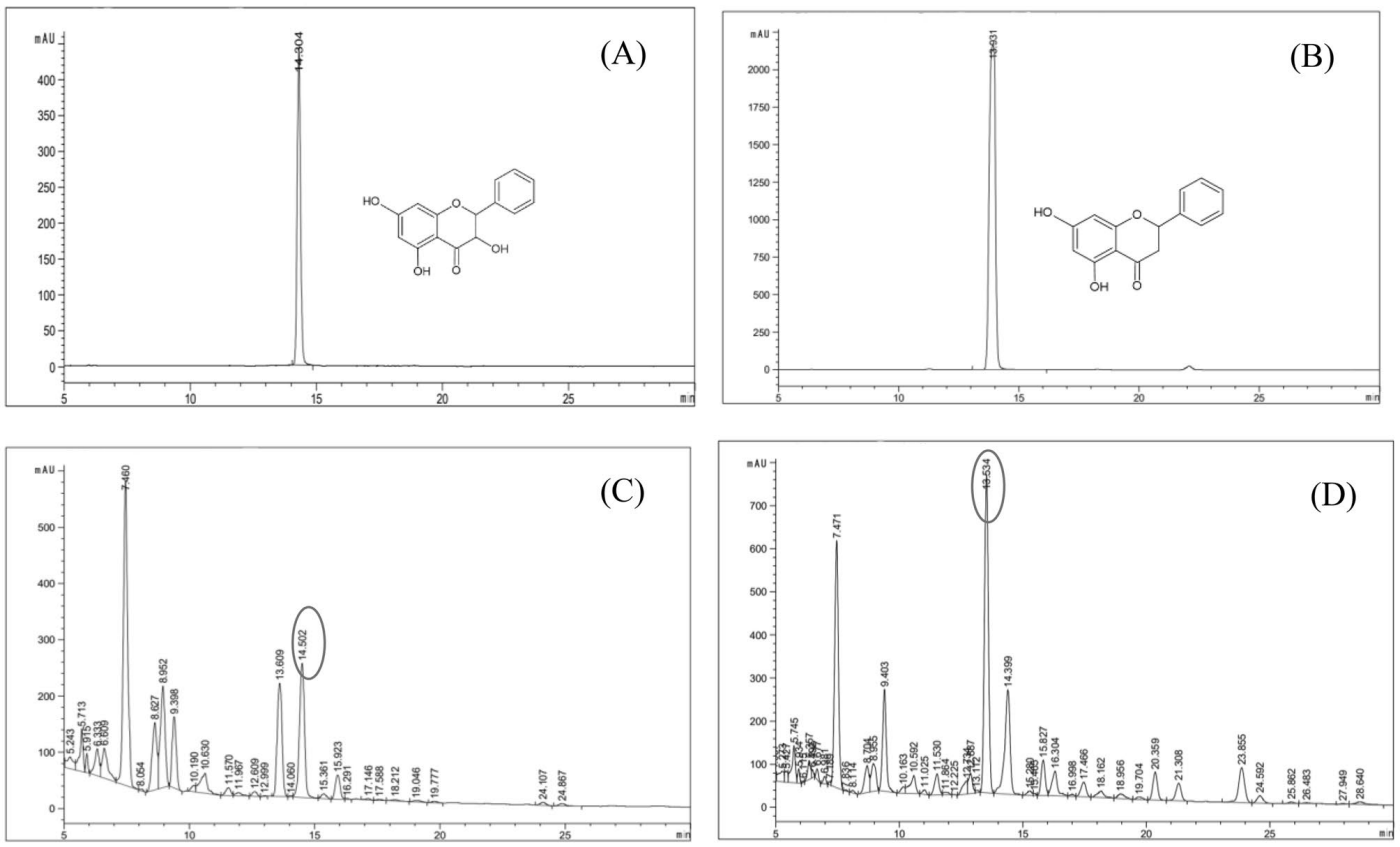
HPLC chromatograms of standard galangin (**A**), chrysin (**B**), SWE obtained at galangin optimal conditions of 200 °C for 20 min (**C**), and chrysin optimal conditions of 170 °C for 20 min (**D**).

Quantitative analysis of flavonoid compounds, such as galangin and chrysin in the SWE extracts was performed using HPLC (Agilent Technologies). The galangin and chrysin concentration were determined using a standard curve at six concentrations (0.5, 0.25, 0.125, 0.0625, 0.03125, and 0.015625 mg/mL). The standard curves for galangin and chrysin were y = 8346x − 61.19 (R^2^ = 0.99), and y = 19301x + 4.05 (R^2^ = 0.99), respectively. The retention times of galangin, and chrysin of SWE extracts were detected at 14.5 min, and chrysin at 13.5 min, respectively (Fig. 2).

**Figure 3:**
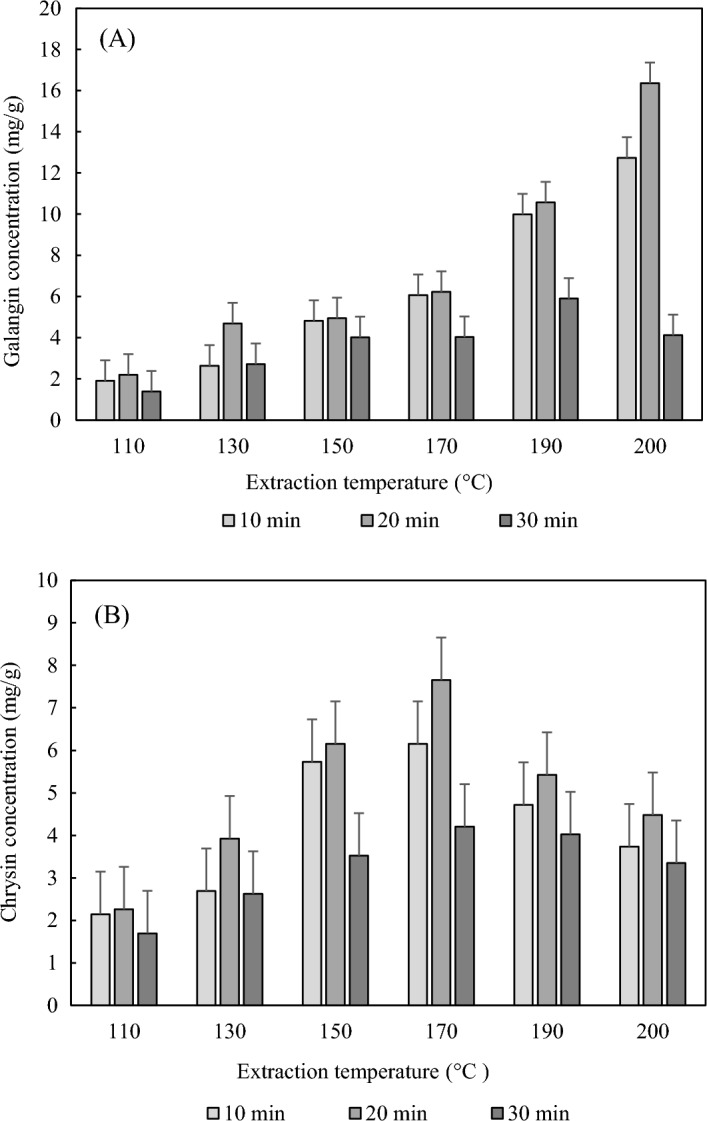
Effects of the extraction temperature and time on the concentration of galangin (**A**) and chrysin (**B**) in SWE extracts. The data represent the mean ± standard deviation (n = 3).

The contents of bioactive compounds, galangin and chrysin, in SWE extracts were measured at extraction temperatures of 110–200 °C for 10–30 min, and the optimal conditions were determined. As shown in Fig. 3A, the optimal conditions of galangin (16.37 ± 0.61 mg/g) in SWE extracts were at 200 °C for 20 min. As the temperature increased from 110 to 170 °C, the content increased slightly. However, at extraction temperatures of 170–190 °C, the concentration of galangin increased rapidly, and a high amount of content was extracted at 200 °C. A study by Cvetanović et al.^24^ also found that galangin was not detected at low temperatures and was extracted only at a high temperature of 210 °C.

As shown in Fig. 3B, the optimal conditions of chrysin (7.66 ± 0.64 mg/g) in SWE extracts were extraction at 170 °C for 20 min. As the temperature increased from 110 to 170 °C, the content increased and then decreased above 170 °C. Above 170 °C, the diversity of A-ring oxygenation results in the formation of numerous natural derivatives of chrysin, such as baicalein, Oroxylin A, and wogonin, resulting in a reduced content of chrysin^25^. Chrysin is a flavone, it has low hydrophilicity and was optimally extracted at a relatively high temperature of 170°C^26^. By identifying the optimal conditions, it was possible to selectively extract the target components.

### TPC

**Figure 4:**
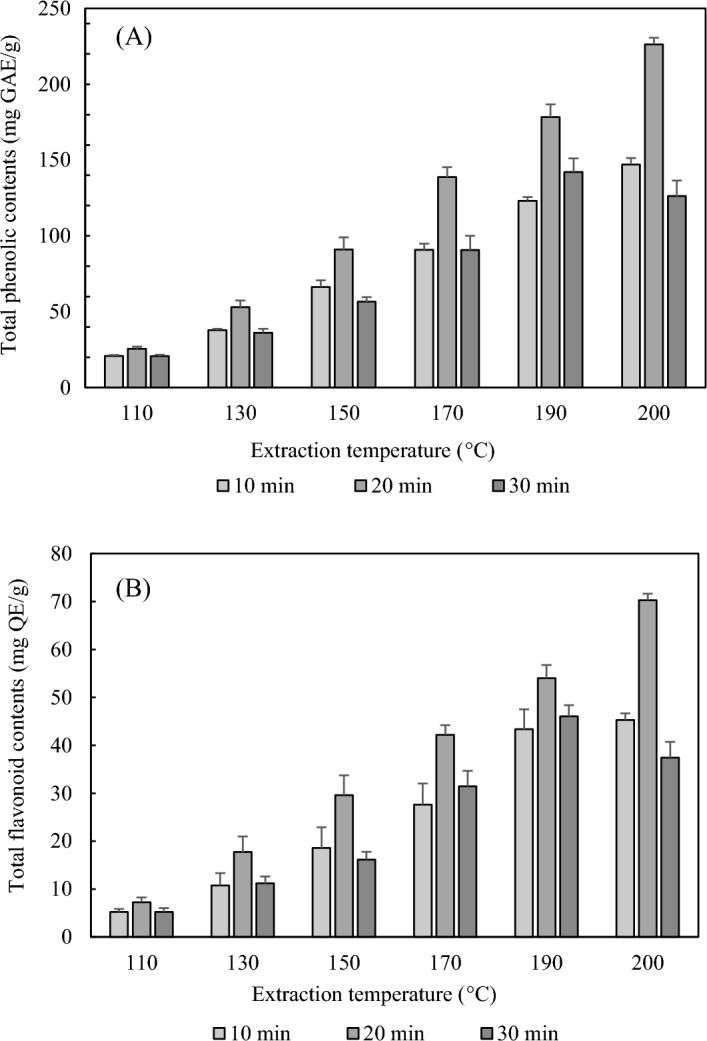
Effects of the extraction temperature and time of SWE on the total phenolic contents (**A**) and flavonoid contents (**B**) in propolis. The data represent mean ± standard deviation (n = 3).

Analysis of the TPC in the SWE extracts was performed using a UV–Vis spectrophotometer, and gallic acid was used as the standard for content calculation. The optimal conditions for phenolics (226.37 ± 4.37 mg GAE/g) from SWE extracts were at 200 °C for 20 min (Fig. 4A). As the extraction temperature increased from 110 to 200 °C at 10 and 20 min, the TPC showed a tendency to increase linearly. The TPC was 20.76 ± 0.85 mg GAE/g at 110 °C, but at 200 °C, this became 226.37 ± 4.37 mg GAE/g, which was more than 10 times higher, showing a significant effect of temperature. In terms of time, at 30 min of extraction time, the content increased as the temperature increased, but it decreased when the temperature reached 200 °C, and the overall content was lower than that of 20 min.

Considering that the optimal temperature condition of TPC was 200 °C, it was deduced that phenolics in SWE extracts were thermally stable^27^. In another study, Similar observations of TPC showed an increase from 80 to 220 °C, and thermal decomposition at 260 °C and 280 °C was observed^28^. In addition, the changes in TPC reduction in the SWE extracts observed after 20 min could be associated with the thermal decomposition of phenolics.

### TFC

Analysis of the TFC in the SWE extracts was performed using a UV–Vis spectrophotometer (Scinco), and quercetin was used as the standard for content calculation. Optimal conditions for flavonoids (70.28 ± 1.33 mg QE/g) from SWE extracts were 200 °C for 20 min (Fig. 4B). As the extraction temperature increased from 110 to 200 °C at 10 and 20 min, the TFC showed a similar trend to that of phenolics. The TFC was 5.23 ± 0.60 mg QE/g at 110 °C, but at 200 °C, this became 70.28 ± 1.32 mg QE/g, which was more than 13 times higher, showing a more significant effect of temperature than phenolics. At 30 min of extraction time, the content increased as the temperature increased, but it decreased at 200 °C, and the overall content was lower than that at 20 min.

Partial hydrolysis of the glycoside derivatives of the flavonols, which can occur at high SWE temperatures, leads to the formation of the aglycone form^29^. Flavonoids, including galangin and chrysin, are present in many aglycone forms and exhibit low solvent polarity and stability at high extraction temperatures during SWE^30^.

### Antioxidant activity assay

To evaluate the antioxidant activities of the SWE extracts, DPPH and ABTS free radical scavenging assays as well as FRAP assays were performed using a UV–Vis spectrophotometer (Scinco). The results of the three SWE assays are shown in Table 1. The highest DPPH and ABTS radical scavenging activity was 88.73 ± 0.58% and 98.86 ± 0.69% at 200 °C for 20 min. The FRAP also showed a similar trend and the highest FRAP was 858.89 ± 11.48 mg/TE g at 200 °C for 20 min. According to the effect of the extraction temperature, the activity increased as the extraction temperature increased at all extraction times of 10, 20, and 30 min. On the other hand, for the effect of extraction time, ABTS free radical scavenging and FRAP increased when time was increased from 10 to 20 min, but these decreased when time was increased to 30 min at 200 °C.

**Table 1:** Effects of the extraction temperature and time of SWE on DPPH radical scavenging activity, ABTS radical scavenging activity, and FRAP in propolis.

Extraction conditions	Antioxidant activity			
Temperature (°C)	Time (min)	DPPH (%)	ABTS (%)	FRAP (mg TE/g)
110	10	62.07 ± 1.37^a^	94.14 ± 0.79^a^	127.19 ± 8.04^ab^
	20	68.10 ± 1.21^c^	96.02 ± 0.43^bcd^	148.96 ± 13.24^bc^
	30	65.15 ± 1.56^b^	94.31 ± 0.55^ab^	122.29 ± 12.48^a^
130	10	68.99 ± 1.28^cd^	94.37 ± 0.95^a^	170.04 ± 1.64^c^
	20	75.06 ± 0.91^f^	96.64 ± 0.79^cde^	244.05 ± 16.35^de^
	30	74.09 ± 1.65^f^	94.82 ± 0.79^a^	229.05 ± 5.63^d^
150	10	71.56 ± 0.84^e^	95.90 ± 0.85^bcd^	255.41 ± 1.63^e^
	20	74.77 ± 0.48^f^	97.04 ± 1.00^de^	395.78 ± 6.15^h^
	30	74.60 ± 0.48^f^	95.51 ± 1.55^abc^	311.84 ± 16.18^f^
170	10	71.22 ± 1.90^de^	96.53 ± 0.77^cde^	357.93 ± 6.02^g^
	20	81.56 ± 1.44^h^	97.90 ± 0.65^ef^	564.65 ± 28.92^k^
	30	77.59 ± 0.44^g^	96.59 ± 0.74^cde^	508.24 ± 23.63^j^
190	10	74.22 ± 1.24^f^	97.33 ± 0.77^de^	457.55 ± 6.64^i^
	20	86.24 ± 0.32^i^	98.86 ± 0.36^f^	715.80 ± 22.39^n^
	30	78.48 ± 0.55^g^	97.67 ± 0.71^ef^	689.69 ± 8.85^m^
200	10	82.28 ± 1.32^h^	97.90 ± 1.03^ef^	582.17 ± 8.39^k^
	20	88.73 ± 0.58^j^	98.86 ± 0.69^f^	858.89 ± 11.48^o^
	30	85.02 ± 1.53^i^	97.61 ± 0.17^ef^	662.81 ± 16.39^l^

Means in a row followed by same superscript letters are not significantly different according to Duncan's test at *p* < 0.05. The data represent the mean ± standard deviation (n = 3).

The SWE temperature at 200 °C has been attributed to the higher extraction yield for antioxidant compounds and the formation of new antioxidant compounds by heat-related reactions, including thermo oxidation, caramelization, and the Maillard reaction^31^. Higher extraction temperatures result in higher amounts of bioactive compounds and higher antioxidant activities. Moreover, in another study, the Maillard reaction product level was the highest under extraction conditions of 200 °C for 20 min^32^.

All antioxidant activities of the SWE extracts showed the same trends as those of TPC and TFC. This meant that by using SWE, phenolics and flavonoids contributed to antioxidant activities, including the radical scavenging activity of SWE extracts; thus, antioxidant activities were found to be closely related to TPC and TFC (Table S1)^33^. There were high correlations (0.886, 0.848, and 0.965, respectively) between TPC and the DPPH, ABTS, and FRAP assay results, and correlations (0.878, 0.867, and 0.960, respectively) between TFC and the DPPH, ABTS, and FRAP assay results. These results suggested that phenolics and flavonoids in propolis may contribute to antioxidant activity in large proportions^34^.

### Comparison with CSE methods

To confirm the efficiency of SWE, the ethanol extraction method, which is the most common method for extracting propolis, was used as a comparative extraction method using the water:ethanol mixture (50:50, 30:70, 0:100, v/v)^35^. Compared with using conventional ethanol extraction (at 25 °C for 24 h), all yields of SWE extracts obtained at 200 °C for 20 min were higher. As shown in Table 2, the TPC (226.37 ± 4.37 mg GAE/g) and TFC (70.28 ± 1.33 mg QE/g) obtained under the optimal SWE conditions at 200 °C for 20 min were 1.3-fold higher than those obtained using 70% ethanol for 24 h (176.28 ± 0.35 mg GAE/g and 56.41 ± 0.65 mg QE/g). All antioxidant activities (88.73 ± 0.58%, 98.86 ± 0.69%, and 858.89 ± 11.48 mg TE/g) obtained under extraction conditions of 200 °C for 20 min were higher than those obtained using 70% ethanol for 24 h (72.74 ± 0.41%, 95.18 ± 0.11%, and 619.51 ± 8.17 mg TE/g).

**Table 2:** Comparison of antioxidative properties obtained using ethanol extraction methods (under temperature/time conditions of 25 °C/24 h) and the SWE method (under temperature/time conditions of 200 °C/20 min) from propolis.

	Extraction solvents			
	SWE	50% ethanol	70% ethanol	100% ethanol
Total phenolics (mg GAE/g)	226.37 ± 4.37^d^	103.07 ± 0.70^a^	176.28 ± 0.35^c^	163.76 ± 1.22^b^
Total flavonoids (mg QE/g)	70.28 ± 1.33^d^	29.52 ± 0.40^a^	56.41 ± 0.65^c^	47.85 ± 0.43^b^
DPPH (%)	88.73 ± 0.58^d^	65.19 ± 0.13^a^	72.74 ± 0.41^b^	66.33 ± 0.33^b^
ABTS (%)	98.86 ± 0.69 ^d^	94.11 ± 0.50^a^	95.18 ± 0.11^c^	95.12 ± 0.13^b^
FRAP (mg TE/g)	858.89 ± 11.48^d^	442.39 ± 5.06^a^	619.51 ± 8.17^c^	565.51 ± 4.87^b^

Means in a row followed by same superscript letters are not significantly different according to Duncan's test at *p* < 0.05. The data represent the mean ± standard deviation (n = 3).

The solids content was measured by weighing the freeze-dried extracts of SWE under various temperature (110–200 °C) and time (10–30 min) conditions and the ethanol extracts after evaporation (Table S2). Overall, the 50, and 70% ethanol extraction yield (243.47 ± 0.01, and 245.10 ± 0.01 mg/0.5 g propolis) were slightly higher than the highest solids content of SWE at 200 °C for 20 min (229.03 ± 0.04 mg/0.5 g propolis). This is because of organic solvents, such as alcohol extract fat-soluble resins and bioactive flavonoids, but fat-soluble ingredients, such as inactive resin materials and beeswax, can also be extracted together. These lipids have low water solubility and low purity of the active ingredients used in food. Despite the higher solid content of 70% ethanol in the above results, all TPC, TFC, and antioxidant activity of SWE extracts were higher than those of 70% ethanol. It was found that SWE filtered out the insoluble components in food and efficiently selectively extracted the bioactive components with high purity. In addition, the SWE time of less than 20 min is much shorter than the 24 h required for CSE. These results confirmed that using only subcritical water as an extraction solvent to extract bioactive compounds from propolis is environmentally friendly, and that SWE, which requires a shorter time, is a more efficient method than CSE methods.

## Conclusions

In this study, propolis was successfully extracted with pure water without an organic solvent by SWE. The optimal extraction conditions for representative flavonoid compounds of propolis, such as galangin and chrysin, and the effects of subcritical extraction temperature and time on phenolic, flavonoid, and antioxidant activities were investigated. The concentrations of galangin (16.37 ± 0.61 mg/g) and chrysin (7.66 ± 0.64 mg/g) in SWE extracts were maximal at 200 °C for 20 min and 170 °C for 20 min separately. Based on these results, when the galangin and chrysin component were extracted as a target, it could be efficiently extracted under optimal SWE conditions. Optimal conditions for phenolics (226.37 ± 4.37 mg GAE/g) and flavonoids (70.28 ± 1.33 mg QE/g) from SWE extracts were 200 °C for 20 min, which were also the optimal conditions for antioxidant activities (88.73 ± 0.58%, 98.86 ± 0.69%, and 858.89 ± 11.48 mg TE/g). In addition, the substitutability and efficiency of the SWE of propolis were confirmed by comparing with CSE with ethanol. The SWE values of TPC and TFC were approximately 1.3 times and 1.2 times higher, and the antioxidant activity was also higher than that of solvent extraction with 70% ethanol. The above results indicated that the SWE technique was not only very effective in extracting phenolics and flavonoids with antioxidant activity selectively from propolis to replace the use of organic solvents, but also has great potential for filtering sticky fat-soluble substances such as viscous resins and waxes.

### Supplementary Information

Supplementary Information.

## Data Availability

All data generated or analysed during this study are included in this published article and its supplementary information files.
