# Supplementary material for: Enhanced extraction of bioactive compounds from propolis (Apis mellifera L.) using subcritical water
Source: Sci Rep. 2023 Sep 12;13:15038. doi: 10.1038/s41598-023-42418-1 (PMC10497595; doi:10.1038/s41598-023-42418-1)
Supplement: Supplementary file 1 — Supplementary Information. [file 41598_2023_42418_MOESM1_ESM.docx]

Table S1. Correlation analyses between antioxidative properties measured by total phenolics, flavonoids, DPPH, ABTS, and FRAP from propolis.

|  | Phenolics | Flavonoids | DPPH | ABTS | FRAP |
| --- | --- | --- | --- | --- | --- |
| Phenolics | 1 |  |  |  |  |
| Flavonoids | 0.985 | 1 |  |  |  |
| DPPH | 0.886 | 0.878 | 1 |  |  |
| ABTS | 0.848 | 0.867 | 0.835 | 1 |  |
| FRAP | 0.965 | 0.960 | 0.918 | 0.840 | 1 |

Correlation is significant at *P* < 0.01.

Table S2. Effect of extraction temperature and time on the solids content of SWE, and CSE from 0.5 g propolis. The data represent mean ± standard deviation (n = 3).

| Extraction method | Extraction conditions | | Solids content (mg/0.5 g propolis) |
| --- | --- | --- | --- |
|  | temperature (°C) | time (min) |  |
| Subcritical water | 110 | 10 | 58.83 ± 0.01 |
|  |  | 20 | 62.00 ± 0.03 |
|  |  | 30 | 51.60 ± 0.01 |
|  | 130 | 10 | 72.67 ± 0.03 |
|  |  | 20 | 86.80 ± 0.02 |
|  |  | 30 | 74.67 ± 0.02 |
|  | 150 | 10 | 104.23 ± 0.02 |
|  |  | 20 | 127.15 ± 0.04 |
|  |  | 30 | 95.63 ± 0.01 |
|  | 170 | 10 | 136.57 ± 0.02 |
|  |  | 20 | 159.20 ± 0.03 |
|  |  | 30 | 146.93 ± 0.04 |
|  | 190 | 10 | 166.60 ± 0.04 |
|  |  | 20 | 193.47 ± 0.04 |
|  |  | 30 | 200.70 ± 0.04 |
|  | 200 | 10 | 190.25 ± 0.03 |
|  |  | 20 | 229.03 ± 0.04 |
|  |  | 30 | 180.00 ± 0.02 |
| 50% ethanol | 25 | 1440 | 243.47± 0.01 |
| 70% ethanol | 25 | 1440 | 245.10 ± 0.01 |
| 100% ethanol | 25 | 1440 | 223.47 ± 0.00 |
